# Abdominal compartment syndrome after endovascular repair for ruptured abdominal aortic aneurysm leads to acute intestinal necrosis: Case report

**DOI:** 10.1097/MD.0000000000005316

**Published:** 2016-11-28

**Authors:** Xiyang Chen, Jichun Zhao, Bin Huang, Ding Yuan, Yi Yang, Yukui Ma

**Affiliations:** Department of Vascular Surgery, West China Hospital of Sichuan University, Chengdu, Sichuan, China.

**Keywords:** abdominal compartment syndrome, endovascular repair, intestinal necrosis, ruptured abdominal aortic aneurysm

## Abstract

**Introduction:**

Abdominal compartment syndrome (ACS) after endovascular repair (EVAR) of rupture abdominal aortic aneurysm (rAAA) is a rare emergency situation, which has a high mortality. However, the progression of ACS is rapid and the diagnosis is usually been delayed, which increase the difficulties in treatment and affect the prognosis. We describe a case of a sever complication (acute intestinal necrosis) resulting from ACS after endovascular repair of rAAA.

**Clinical Finding:**

An elderly man, 81 years old, complained a sudden lower abdominal and back pain without any predisposing cause. He had a history of hypertension for 20 years without any regular anti-hypertensive therapy. Physical Examination revealed that the blood pressure was 89/54 mmHg, pulse was 120/min, oxygen saturation was 91%. The abdominal ultrasound and the CTA (computed tomography angiography) scan revealed a rAAA. Emergency EVAR under general anesthesia was performed for this patient.

**Diagnosis:**

Fourteen hours after endovascular repair, sudden decreased of blood pressure (70/50 mmHg) and oxygen saturation (70%) was observed. ACS or bleeding of retroperitoneal space was diagnosed.

**Interventions:**

Abdominal laparotomy was immediately performed. ACS was verified and a severe complication (acute intestinal necrosis) was observed, intestinal resection was performed for this patient.

**Outcomes:**

Unfortunately, this patient died after operation because of multi-organ failure in a very short period, which is very rare regarding to this condition. Surgical pathology, diagnosis and management were discussed.

**Conclusion:**

ACS was occurred with a severe complication (acute intestinal necrosis) in a very short period, which is very rare regarding to this condition after EVAR, it reminds us the severe result of ACS and more methods to prevent it happened after surgical management.

## 1 Introduction

Abdominal compartment syndrome (ACS) is a well-documented complication which leads to multiple organ failure and postoperative death in rAAA patients undergoing open repair. In contrast to open repair, to date, limited literature was reported regarding the occurrence of ACS after EVAR of rAAA; we will discuss a case of acute intestinal necrosis caused by ACS after EVAR of rAAA.

## 2 Presenting concerns

The subject of this report is an 81-year old, smoking, drinking, married, male. He had a history of hypertension for 20 years without any regular antihypertensive therapy. Ordinary time, the blood pressure for this patient waves around 150/80 mm Hg to 180/100 mm Hg. Coronary heart disease was diagnosed through coronary arteriongraphy for the symptom of chest pain after exercise in another hospital (the details condition of coronary arteries were unknown). Stent was implanted at that time and this patient did not complaint about chest pain after the procedure. Antiplatelet was given for this patient, but he did not do any physical examination again to check the patency of stent and blood pressure.

## 3 Clinical findings

This patient complained of an acute lower abdominal and back pain for 8 hours in January 2015 was immediately sent to our emergency department. ECG (electrocardiogram) monitoring was performed and oxygen was immediately given. Physical examination revealed that the blood pressure was 89/54 mm Hg, pulse was 120/min, oxygen saturation was 91%. A pulsatile mass could be palpated with a severe lower abdominal tenderness. Blood values at admission were: Hb = 11.5 g/dL; WBC = 11.2 × 10^9^/l; PLT = 69 × 10^9^/l; INR = 1.13, renal profile, was normal.

## 4 Timeline

At 14:00 pm, Jan 3, 2015, this patient complained about a sudden lower abdominal and back pain and transferred to our emergency department at 14:40 pm. After performing abdominal CTA, ultrasound and blood test at 15:30 pm, the diagnosis of rAAA was made. Emergency endovascular repair for this patient was performed at 16:00 pm, the operation was successfully finished at 17:00 pm, and the patient was sent to ICU (intensive care unit). A sudden decreased of blood pressure and oxygen saturation was observed at 7:00 am, Jan 4, 2015, cross-check the blood test and physical sign was performed, the diagnosis of ACS or bleeding was considered, and the emergency abdominal laparotomy was performed at 9:20 am, after the operation, the general situation for this patient worsened gradually within 3 to 4 hours. This patient died because of multiorgan failure at 16:10 am.

## 5 Diagnostic focus and assessment

**Figure 1:**
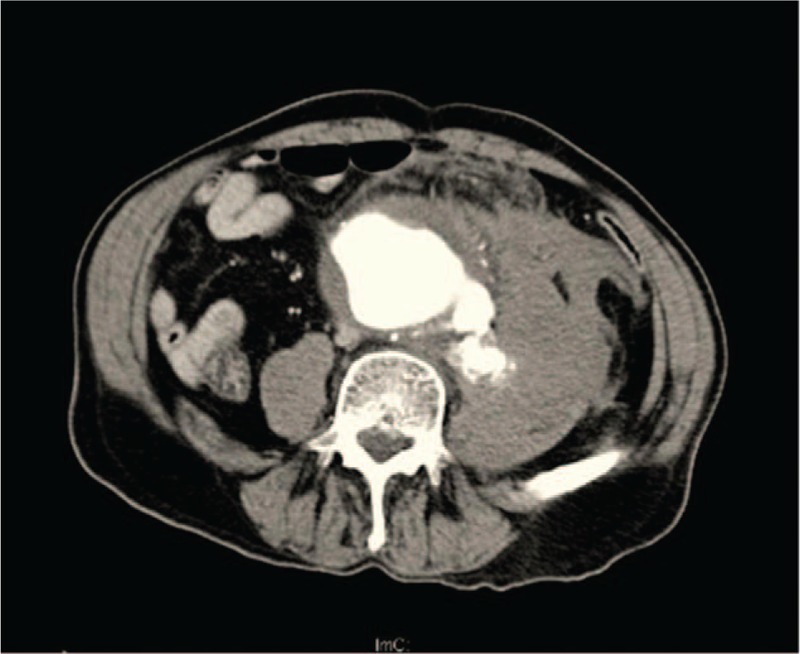
CT scan shows rAAA with huge hematoma in the left side of retroperitoneal space. CT = computed tomography, rAAA = ruptured abdominal aortic aneurysm.

Abdominal ultrasound and CTA scan were revealed an rAAA with maximum diameter of 8.74 cm, and a huge hematoma can be seen in the left retroperitoneal space (Fig. 1). Combined with his admission blood values, vital signs, and history of hypertension, the diagnosis of rAAA with hemorrhagic shock was verified. Considering his age, history of coronary artery disease, and unstable vital signs, the risk of open repair for this patient was too high and the prognosis trends to be worse than EVAR because the characteristic of minimal invasive of EVAR. Even though, due to the poor basic condition for this patient, the risk of EVAR was still high and the prognosis for this patients remained uncertain.

## 6 Therapeutic focus and assessment

**Figure 2:**
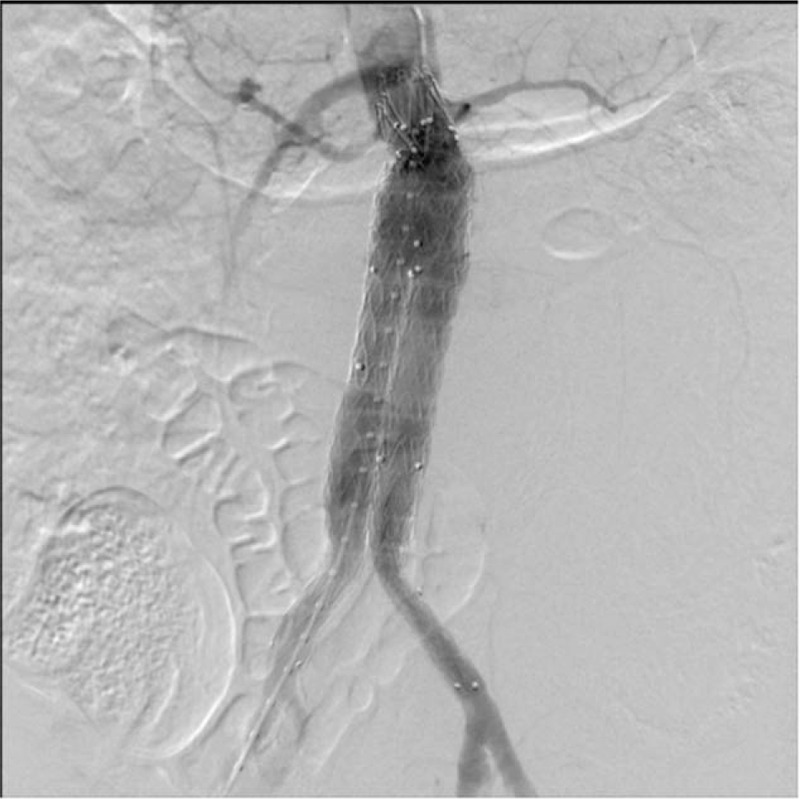
Angiography after EVAR, no endoleak was observed. EVAR = endovascular aneurysm repair.

Considering the hemorrhagic shock and poor basic condition of these patients, the emergency EVAR was immediately performed under general anesthesia. Angiography verified the contrast outflow from left side of aneurysm and there was calcification of abdominal aorta wall with an angulated aneurysmal neck. A Medtronic bifurcated stent was implanted through bilateral common femoral artery and implanted just below the orifice of renal artery to cover the aneurysm. Re-examination of angiograph showed that stent was accurately located just below renal artery with aneurysm completely covered by stent, there was no endoleak observed, whereas the vital signs for this patient become stable (blood pressure:105/70 mm Hg, pulse: 100/min, oxygen saturation: 96%). The procedure of EVAR was successful (Fig. 2).

## 7 Follow-up and outcomes

**Figure 3:**
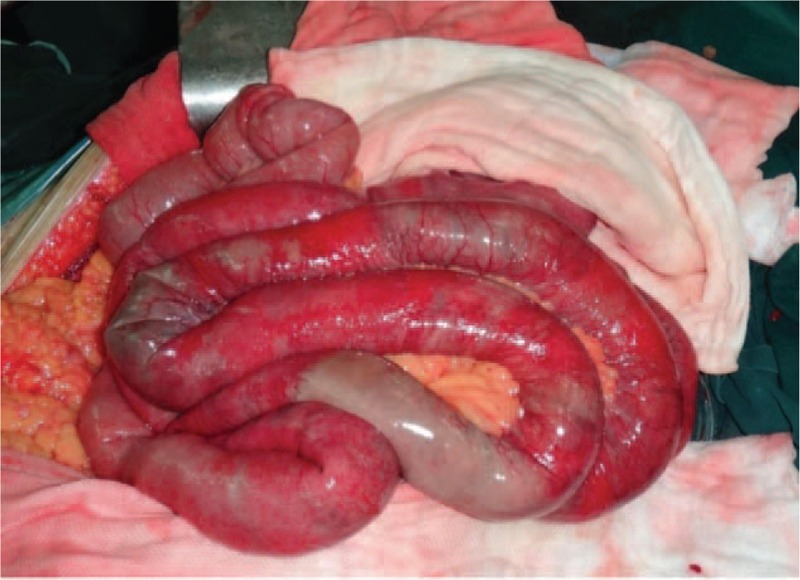
Intraoperative appearance of intestinal necrosis.

**Figure 4:**
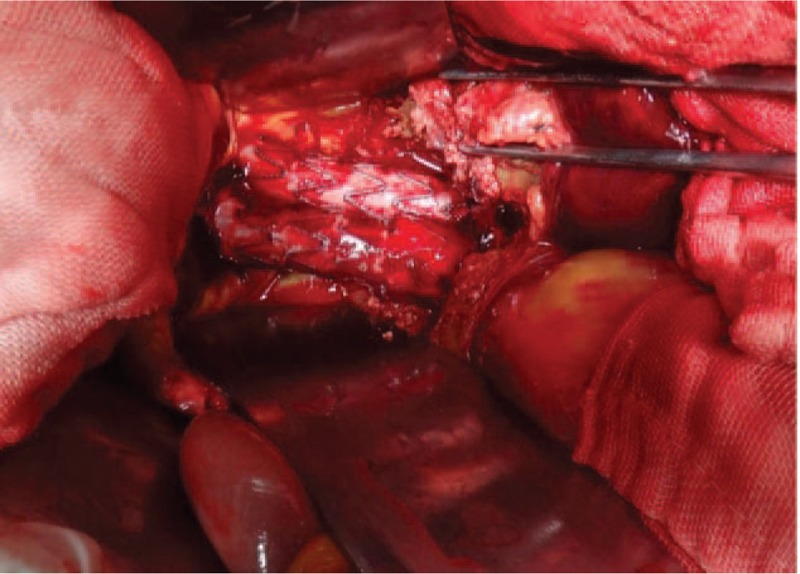
No bleeding or endoleak observed in aneurysmal sac.

After EVAR, this patient was sent to ICU with mechanically ventilation for postoperative care. Fourteen hours after EVAR, a sudden decrease of blood pressure (70/50 mm Hg) and oxygen saturation was observed (70%). At that time, this patient was still with mechanically ventilation and the patient was still unconscious. Physical examination revealed a distended abdomen with increased tension and weak bowel sound. Emergency blood values showed: Hb = 7.1 g/dL; WBC = 25.6 × 10^9^/L; PLT = 37 × 10^9^/L; INR = 1.43; ALT = 1097IU/L, AST = 1283IU/L, ALB = 23.7 g/L, Glu = 6.03 mmol/L, BUN = 12.13 mmol/L, Crea = 270 μmol/L, Na^+^ = 138.4 mmol/L, K^+^ = 6.12 mmol/L, Cl^−^ = 99.5 mmol/L, Ca^2+^ = 1.81 mmol/L, Mg^2+^ = 1.03 mmol/L, Lac = 12.2 mmol/L, PO_2_ = 68 mm Hg, PCO_2_ = 35.3 mm Hg, PH = 7.30. Blood infusion and correction of metabolic acidosis were performed immediately and re-examination of blood value 1.5 hours later showed: Hb = 5.1 g/dL; WBC = 35.1 × 109/L; PLT = 30 × 109/L; INR = 1.51; ALT = 1061IU/L, AST = 1083IU/L, ALB = 21.7 g/L, Glu = 5.58 mmol/L, BUN = 10.13 mmol/L, Crea = 281 μmol/L, Na^+^ = 138.1 mmol/L, K^+^ = 5.62 mmol/L, Cl^−^ = 94.5 mmol/L, Ca^2+^ = 1.91 mmol/L, Mg^2+^ = 1.13 mmol/L, Lac = 10.1 mmol/L, PO_2_ = 57 mm Hg, PCO_2_ = 38.3 mm Hg, PH = 7.377. The 2 possibilities of diagnosis were considered for this patient: ACS or bleeding of retroperitoneal space. Abdominal laparotomy was performed immediately to detect the reason for change in condition. Intraoperative exploration revealed that there was change in color of the intestinal wall ranging from distal of jejunum to proximal of ileum, the total length of the intestinal involvement was about 140 cm, no movement can be observed in this ischemia intestinal tube, no mesenterial vascular occlusion was observed (Fig. 3). A hematoma can be seen in the left retroperitoneal space without bleeding. There was no bleeding or endoleak in the aneurysmal sac (Fig. 4). The intestinal tube section did not change after warming with saline solution for 30 minutes, intestinal resection was performed finally for this patient, the total length of intestinal tube resected was about 160 cm (100 cm of jejunum and 60 cm of ileum separately), the ileocecal part was maintained for this patient. After operation, this patient was sent to ICU department with mechanically ventilation. Vital signs showed that blood pressure was 68/42 mm Hg under dopamine and noradrenalin, heart rate was 100/min, oxygen saturation was 87%. Re-examination of blood values showed: Hb = 7.9 g/dL; WBC = 8.46 × 10^9^/L; PLT = 30 × 10^9^/L; INR = 3.83; ALT = 1078IU/L, AST = 2527IU/L, ALB = 10.7 g/L, Glu = 3.03 mmol/l, BUN = 16.5mmol/L, Crea = 320 μmol/L, Na^+^ = 146.6 mmol/L, K^+^ = 4.51 mmol/L, Cl^−^ = 102.3 mmol/L, Ca^2+^ = 3.80 mmol/L, Mg^2+^ = 0.83 mmol/L, Lac = 11.2 mmol/L, PO_2_ = 78 mm Hg, PCO_2_ = 41.4 mm Hg, PH = 7.366. A series of medical treatment including blood perfusion, anti-inflammation, correction of acidosis and hyperkalemia, mechanical ventilation were given to this patient. But the respiratory, liver and renal function deteriorated within 4 hours. The last blood values of this patient showed: Hb = 6.1 g/dL; WBC = 7.46 × 10^9^/L; PLT = 21 × 10^9^/L; INR = 5.92; ALT = 746IU/L, AST = 2163IU/L, ALB = 9.0 g/L, Glu = 3.22 mmol/L, BUN = 15.6 mmol/L, Crea = 323 μmol/L, Na^+^ = 146.9 mmol/L, K^+^ = 5.66 mmol/L, Cl^−^ = 102.9 mmol/L, Ca^2+^ = 1.95 mmol/L, Mg^2+^ = 1.25 mmol/L, Lac = 13.6 mmol/L, PO_2_ = 55 mm Hg, PCO_2_ = 66.7 mm Hg, PH = 7.026, the blood pressure maintained about 65/40 mm Hg with dopamine and noradrenalin pumping, heart rate was 87/min, and oxygen saturation was about 87%. Finally, this patient eventually died due to the multiorgan failure in ICU department.

## 8 Discussion

ACS is defined as a sustained intra-abdominal pressure(IAP) >20 mm Hg (with or without an abdominal perfusion pressure <60 mm Hg) that is associated with new organ dysfunction/failure.^[1]^ Although ACS is nowadays increasingly recognized for EVAR of rAAA by vascular surgeons,^[2,3]^ reports are scare to the occurrence of ACS after EVAR of rAAA.

According to the literature,^[4]^ the incidence of ACS after EVAR of rAAA is ∼8%, although it is said that there is no significant relationship between ACS and death, half of patients who developed ACS after EVAR of rAAA are likely to die. Compare with diagnosis of ACS after EVAR, the ideal management with ACS after EVAR is a more complex matter. When and how to deal with the rAAA patients who suffer from ACS after EVAR is still under controversial. Some author suggests to evaluate the potential risk factors to predict the chance for ACS after EVAR of rAAA, Mehta^[5]^ documented that (1) the need for an aortic occlusion balloon, (2) the presence of severe coagulopathy, (3) massive transfusion requirements, and (4) the emergent conversion of modular bifurcated stent grafts to aortouniiliac devices were all associated with the development of ACS. But as we know, patients with rAAA are more or less accord with conditions above; in our case, this patient reached the condition 1 to 3, and another systemic review^[4]^ did not identify any risk factors for ACS after EVAR of rAAA. Once ACS is diagnosed, medical management is recommended first including respiratory support (intubation, ventilation, and muscle-relaxation), cardiac support (increase cardiac output), and temporary hemofiltration; if no improvement is observed with medical management, surgical management should be performed. To date, surgical management includes urgent decompression laparotomy, assessment for intestinal ischemia, evacuation of hematoma, temporary abdominal closure, and delayed repair.^[6–8]^ But according to our case, the development of ACS in patients with rAAA is very rapid and threatening, it took no more than 2 hours for us from awareness of ACS to intervention, but the result is still disastrous. Some doctors advocated early prophylactic surgical management for patients who were with high risk in ACS, but advantages of minimally invasive of EVAR was gone and it will increase the risk of infection and bleeding. There was a consensus that routine assessment of intra-abdominal pressure (IAP) was recommended after EVAR of rAAA, once IAP>20 mm Hg,^[9]^ surgeons should be aware of development of ACS. For this case, acute intestinal necrosis was rarely reported due to ACS after EVAR of rAAA, but it reminds us the severe result for ACS and more evidence should be offered to evaluate the risk factor of ACS for rAAA and identify the accurate time for surgical management.

## References

[1] Bjorck M Wanhainen A Management of abdominal compartment syndrome and the open abdomen. Eur J Vasc Endovasc Surg 2014;47:279–87. 24447530 10.1016/j.ejvs.2013.12.014

[2] Mayer D Rancic Z Veith FJ How to diagnose and treat abdominal compartment syndrome after endovascular and open repair of ruptured abdominal aortic aneurysms. J Cardiovasc Surg 2014;55:179–92. 24670826

[3] Rubenstein C Bietz G Davenport DL Abdominal compartment syndrome associated with endovascular and open repair of ruptured abdominal aortic aneurysms. J Vasc Surg 2015;61:648–54. 25499708 10.1016/j.jvs.2014.10.011

[4] Karkos CD Menexes GC Patelis N A systematic review and meta-analysis of abdominal compartment syndrome after endovascular repair of ruptured abdominal aortic aneurysms. J Vasc Surg 2014;59:829–42. 24439324 10.1016/j.jvs.2013.11.085

[5] Mehta M Darling RC 3rd Roddy SP Factors associated with abdominal compartment syndrome complicating endovascular repair of ruptured abdominal aortic aneurysms. J Vasc Surg 2005;42:1047–51. 16376190 10.1016/j.jvs.2005.08.033

[6] Mayer D Pfammatter T Rancic Z 10 years of emergency endovascular aneurysm repair for ruptured abdominal aortoiliac aneurysms: lessons learned. Ann Surg 2009;249:510–5. 19247042 10.1097/SLA.0b013e31819a8b65

[7] Horer T Skoog P Pirouzram A Tissue plasminogen activator-assisted hematoma evacuation to relieve abdominal compartment syndrome after endovascular repair of ruptured abdominal aortic aneurysm. J Endovasc Ther 2012;19:144–8. 22545876 10.1583/11-3699.1

[8] Djavani Gidlund K Wanhainen A Bjorck M Intra-abdominal hypertension and abdominal compartment syndrome after endovascular repair of ruptured abdominal aortic aneurysm. Eur J Vasc Endovasc Surg 2011;41:742–7. 21411345 10.1016/j.ejvs.2011.02.021

[9] Horer TM Skoog P Norgren L Intra-peritoneal microdialysis and intra-abdominal pressure after endovascular repair of ruptured aortic aneurysms. Eur J Vasc Endovasc Surg 2013;45:596–606. 23540804 10.1016/j.ejvs.2013.03.002

